# Nutritional status and activities of daily living in patients with Parkinson’s disease

**DOI:** 10.1371/journal.pone.0246329

**Published:** 2021-02-02

**Authors:** Tomohiko Nagano, Tatsuyuki Kakuma, Yuichi Umezu, Takashi Yanagawa

**Affiliations:** 1 Kokura Rehabilitation Hospital, Kitakyushu, Fukuoka, Japan; 2 Biostatistics Center, Kurume University, Kurume, Fukuoka, Japan; German Centre for Neurodegenerative Diseases Site Munich: Deutsches Zentrum fur Neurodegenerative Erkrankungen Standort Munchen, GERMANY

## Abstract

Patients with Parkinson’s disease are often frail and likely to be malnourished. Several studies have reported the adverse effects of malnutrition on functional outcomes; however, the association between nutritional status and activities of daily living is unclear among patients with Parkinson’s disease. This study aimed to investigate the relationship between nutritional status and activities of daily living in patients with Parkinson’s disease. We conducted a retrospective cohort study with the data of 124 patients who were consecutively admitted to a rehabilitation hospital in Japan, among whom the data of 61 patients were included in the analyses. The Controlling Nutritional Status score was used to measure the nutritional status of the participants, and the motor subdomain of the Functional Independence Measure was used to assess the activities of daily living. Piecewise linear mixed-effects models were fitted to the data after adjusting for confounding factors. A poor nutritional status (i.e., Controlling Nutritional Status score >3) was significantly associated with a poor Functional Independence Measure gain, which was defined as difference in the score values of the Functional Independence Measures between discharge and admission. Our findings could aid in developing nutritional intervention programs for patients with Parkinson’s disease by employing the Controlling Nutritional Status score to improve their activities of daily living.

## Introduction

Parkinson’s disease (PD) is a progressive disease often complicated by dysphagia when symptoms progress and may cause decreased daily dietary intake, weight loss, and malnutrition. A systematic review of nutrition in patients with PD reported that the rate of malnutrition was 0%–24%, and the rate of being at risk of malnutrition was 3%–60% [1].

Nutritional status is important to improve activities of daily living (ADL). Visvanathan et al. [2] and Thomas et al. [3] reported that patients with malnutrition during hospitalization have a greater number of acute events, require more long-term medical treatment, and have a lower likelihood of being discharged to home. Furthermore, improved nutritional status was related to improved ADL at discharge from the hospital in patients with hip fractures [4] and strokes [5, 6].

Nutritional management for patients with PD is considered particularly important. However, to the best of our knowledge, no reports have focused on the nutritional status and ADL in patients with PD. This could be due to lack of appropriate methods for evaluating nutritional status in this patient population. Since the population of patients with PD is too heterogeneous, it is not easy to find a good measuring tool that can be applied to all. However, various measures have been developed for nutritional assessment in older people such as the Mini Nutritional Assessment [7, 8], Malnutritional Universal Screening Tool [9], body mass index (BMI) [10, 11], weight loss [12, 13], and skeletal muscle mass [14] to detect malnutrition. In this study, we employed the Controlling Nutritional Status (CONUT) score, which we found useful for evaluating the nutritional status of patients with PD.

We conducted a retrospective cohort study with data of 124 consecutive patients who were admitted to a rehabilitation hospital in Japan, among whom data of 61 patients were included in the analyses. The CONUT score was used to measure the nutritional status of the participants, and the motor subdomain of the Functional Independence Measure (FIM) was used to assess the ADL. Based on the rationale that “the ADL is stable to some point, then decreases with an increase in malnutrition,” we fitted piecewise linear mixed-effects models to the data after adjusting for confounding factors, in addition to fitting a linear mixed-effects model. A poor nutritional status (i.e., CONUT score >3) was found to be significantly associated with a poor FIM gain, which was defined as the difference in the score values of the FIM between discharge and admission.

## Materials and methods

### Patients

**Fig 1:**
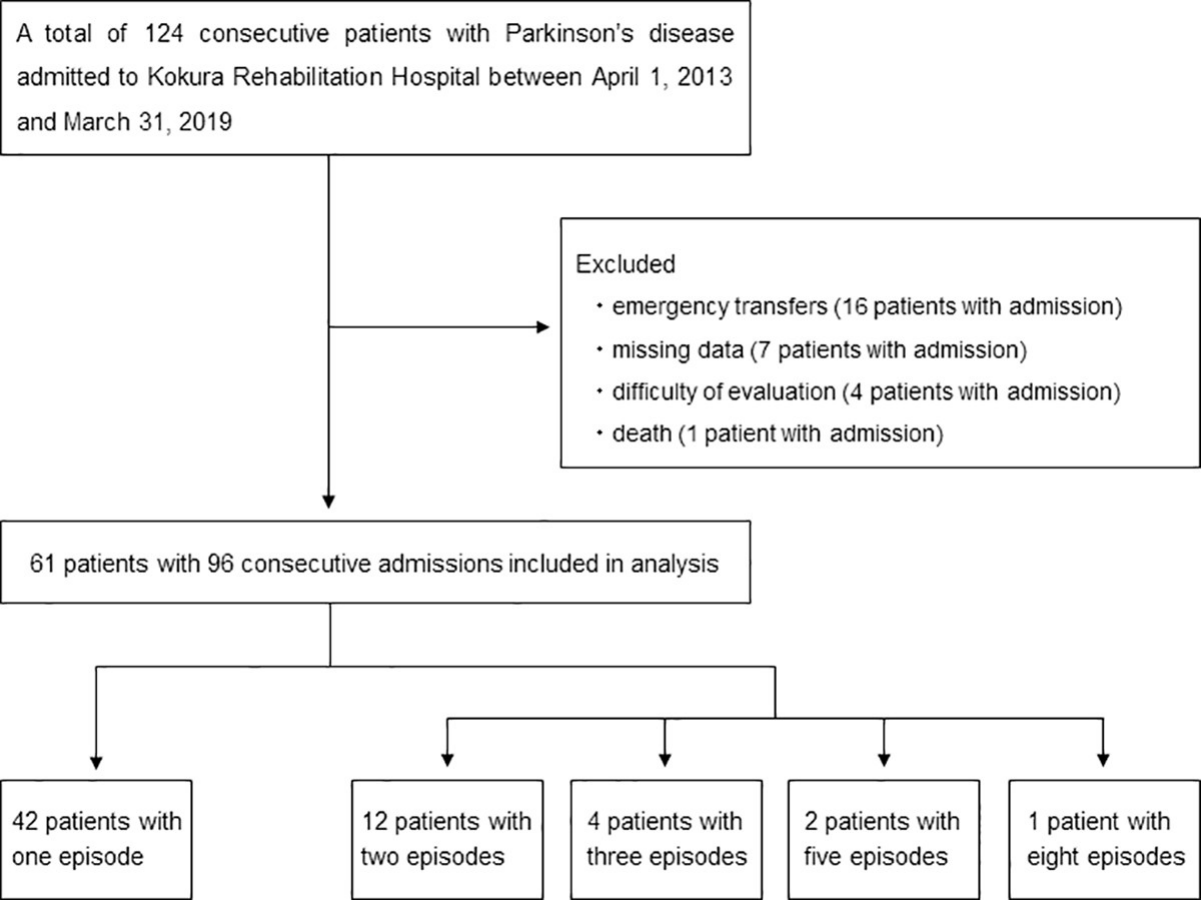
Flowchart for patient selection.

A total of 124 consecutive patients with PD who were admitted to Kokura Rehabilitation Hospital between April 1, 2013, and March 31, 2019, were enrolled in this study. Due to emergency transfer, missing data, difficulty of evaluation, and death, finally, 61 patients with 96 consecutive admissions were selected for data analysis. Notably, some patients were admitted more than once during the study period; thus, repeated measurements obtained from one patient were correlated. A detailed flowchart of patient selection is depicted in Fig 1.

### Ethical considerations

The study protocol conformed to the ethical guidelines of the Declaration of Helsinki and was approved by the ethics committee of Kokura Rehabilitation Hospital (No.10001) and Kurume University (No.19073). The ethics committee waived the requirement for informed consent because this was a retrospective study. Additionally, we provided information regarding the study to all patients concerned, while giving them an opt-out option, which allowed patients to withdraw from the study at any time. For the protection of patient privacy, patient data were collected with unlinkable anonymization and saved in a password-protected storage medium for research use only. The data set is available upon request to the first author of this paper.

### Nutritional status

The nutritional status was diagnosed using the CONUT method [15]. The CONUT method is a method of scoring albumin (Alb), total lymphocyte count (TLC), and total cholesterol (T-cho) values. Scoring methods of these items are illustrated in Table 1.

**Table 1:** Scoring methods of Alb, TLC, and T-cho values.

Alb (g/dl)	≥3.50	3.00–3.49	2.50–2.99	<2.50
Score	0	2	4	6
TLC (/μL)	≥1600	1200–1599	800–1199	<800
Score	0	1	2	3
T-cho (mg/dl)	≥180	140–179	100–139	<100
Score	0	1	2	3

Alb, albumin; TLC, total lymphocyte count; T-cho, total cholesterol.

The CONUT score was computed using the following formula:

CONUT score = (Alb score) + (TLC score) + (T-cho score).

CONUT scores of 0–1, 2–4, 5–8, and 9–12 are considered to indicate normal nutritional status, light malnourishment, moderate malnourishment, and severe malnourishment, respectively [15].

### Activities of daily living

ADLs were assessed using the FIM [16]. The FIM is one of the most common measures of ADL and includes 13 lower-order items regarding motor function and five lower-order items regarding cognitive function. Each item is graded on a scale from 1 (total assistance) to 7 (complete independence). The FIM scores on admission and at discharge were assessed by a team of physical therapists, occupational therapists, and nurses. The motor FIM score is the total score of the motor function, ranging from 13 to 91 points. The primary outcome was FIM gain, which was defined as the difference in the motor FIM scores at discharge and admission.

### Data

The following variables were reviewed from the medical records: age, sex, number of days of hospitalization, route of hospitalization, Alb (g/dl), TLC (/μL), T-cho (mg/dL), BMI, PD severity, dysphagia, grip strength, adjusting anti-Parkinsonian drugs, weight on admission and discharge, and the FIM. The CONUT score was calculated from the Alb, TLC, and T-cho values. The weight loss rate was defined as the difference between the weight at discharge and admission, divided by the weight on admission. The Hoehn and Yahr stage (HY stage) [17] and the Food Intake LEVEL Scale [18] were also considered. The HY stage was used to classify patients with PD into two states, severe (HY stage 4–5) or not severe (HY stage 1–3), and the Food Intake LEVEL Scale was employed to classify dysphagia as “Yes” when it was ≤9 and “No” when it was 10.

### Statistical analysis

Because randomization was not employed in the study design, study findings could be easily influenced by confounding factors. We identified confounding factors at the beginning, based on the data, as follows: First, variables associated with FIM gain at a significance level of 10% were selected. Then, among these variables, those that were found to have a significant association with the CONUT score, at a significance level of 10%, were identified as confounding factors.

A mixed-effects model was employed to deal with the correlated measurements from a single patient due to repeated admissions. Also, linear and piecewise linear models with one knot were considered, and they were combined with the mixed-effects model. The combined models were fitted to the data after adjusting for confounders, and in the case of piecewise linear model, the model was repeatedly fitted to the data by changing the values of the knot in the CONUT scores. The model that achieved the smallest Akaike’s Information Criteria was selected as the best model. Details of the models and model fitting process are described in the supporting information section (S1 File). Statistical analysis was performed using SAS software version 9.4 (SAS Institute Inc., Cary, NC, USA).

## Results

### Baseline characteristics of the patients

Table 2 shows the baseline characteristics of the patients. The values in the table are presented as the median and interquartile range for numerical variables and as numbers and percentages (%) for categorical variables. Age, BMI, CONUT score, and grip strength values presented in the table were obtained on admission. The median age was 76.5 years. Of the 96 admissions, 53 (55.2%) were male, 53 (55.2%) patients with PD were categorized as severe, and 55 (57.3%) patients had dysphagia.

**Table 2:** Baseline characteristics of the patients.

Background factors	Median (IQR)
Age (years)	76.5 (70, 79.7)
Number of days of hospitalization (days)	80 (49.2, 136.2)
Laboratory data	
Albumin (g/dL)	3.6 (3.2, 3.9)
Total lymphocyte count (/μL)	1,581 (1,299.4, 1953.6)
Total cholesterol (mg/dL)	161 (144, 179.7)
BMI (kg/m^2^)	19.6 (17.3, 22.7)
Weight loss rate (%)	0.7 (-5.3, 3.3)
Grip strength (kg)	17.9 (12.9, 26.8)
FIM (score)	
Total	79.5 (34, 100.7)
Motor	49.5 (15.7, 68)
Cognitive	29 (14.2, 35)
Gain	6 (0, 13.7)
Background factors	N. (%)
Male sex	53 (55.2)
Route of hospitalization	
Acute care hospital	59 (61.5)
Home	34 (35.4)
Others	3 (3.1)
CONUT score	
0–1	27 (28.1)
2–4	46 (47.9)
5–8	23 (24.0)
9–12	0 (0)
PD severity	
HY stage 1–3	43 (44.8)
HY stage 4–5	53 (55.2)
Dysphagia	
No	41 (42.7)
Yes	55 (57.3)
Adjusting anti-Parkinsonian drugs	
No	65 (67.7)
Yes	31 (32.3)

IQR, interquartile range; BMI, body mass index; FIM, Functional Independence Measure.

CONUT, Controlling Nutritional Status; PD, Parkinson’s disease; HY stage, Hoehn and Yahr stage.

### Identification of confounding factors

**Fig 2:**
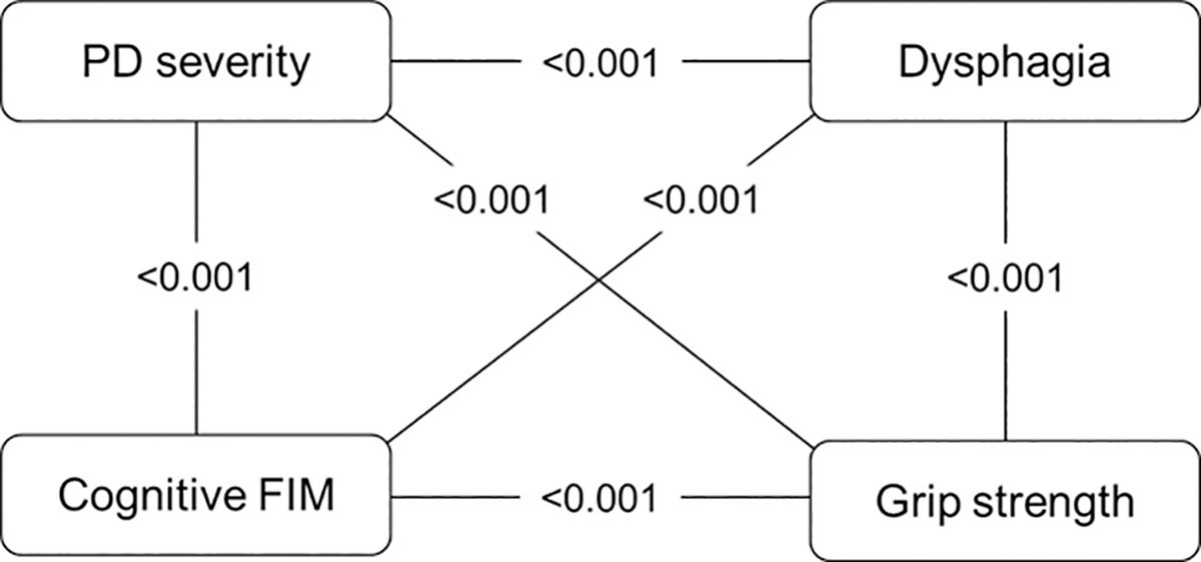
Relationships of the four confounding factors. The values in the figure represent the p-value between the two variables. PD, Parkinson’s disease; FIM, Functional Independence Measure.

Variables that were significantly associated with the FIM gain, at the 10% significance level, included PD severity (p<0.001), dysphagia (p = 0.005), cognitive FIM (p = 0.089), and grip strength (p = 0.080). Furthermore, variables that were significantly associated with the CONUT score, at a 10% level of significance, included PD severity (p = 0.011), dysphagia (p = 0.001), cognitive FIM (p = 0.001), and grip strength (p<0.001). Thus, PD severity, dysphagia, cognitive FIM, and grip strength were identified as confounding factors. Fig 2 shows the relationship of the four confounding factors, and these factors were strongly related to each other. We selected PD severity as a representative confounder.

### Relationship between the CONUT score and FIM gain

The piecewise linear model with knot = 3 was selected as the best fitted model. The estimated parameters of the best fitted model are summarized in Table 3. Details of the model formula and process of estimating parameters in the formula are presented in the (S1 File). From the table, the relationship between the FIM gain and the CONUT score, after adjusting for age, sex, and PD severity, is given as follows:

If the CONUT score is ≤3:

FIM gain = 5.864 + 1.465 × CONUT score + 0.067 × age + 1.383 × sex − 8.948 × PD severity,

and if the CONUT score is >3:

FIM gain = 16.421− 2.054 × CONUT score + 0.067 × age + 1.383 × sex − 8.948 × PD severity,

where sex = $\left\{ \begin{array}{c} 0:\;male\\ 1:\;female \end{array}\right.$, PD severity = $\left\{ \begin{array}{c} 0:\;not\;severe\\ 1:\;severe \end{array}\right.$.

**Table 3:** Estimated parameters and knot in the best fitted model.

Parameter	Coefficient	*p*-value	95%CI	
Intercept	5.864	0.602	-16.528	28.257
CONUT score ≤3	1.465	0.183	-0.729	3.659
CONUT score >3	-2.054	0.026	-3.847	-0.260
Age	0.067	0.665	-0.248	0.383
Sex	1.383	0.549	-3.277	6.044
PD severity	-8.948	<0.001	-13.360	-4.535
Knot of piecewise linear model	3			
$\sigma_{b}^{2}$ (between subject variance)	32.898			
$\sigma_{\varepsilon }^{2}$ (within subject variance)	61.760			

CI, confidence interval; CONUT, Controlling Nutritional Status; PD, Parkinson’s disease.

**Fig 3:**
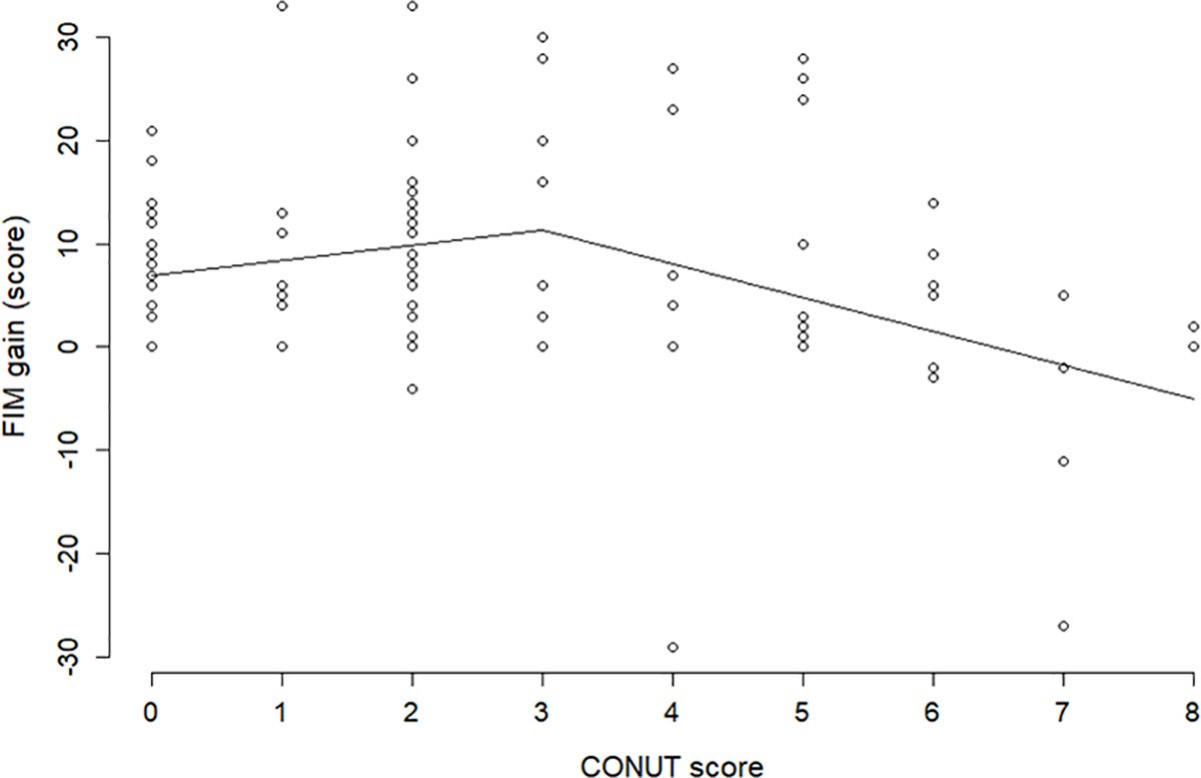
Estimated relationship of the CONUT score and FIM gain. Mean values are assigned respectively to age, sex (0: male, 1: female), and PD severity (0: not severe, 1: severe) in the best fitted model. FIM, Functional Independence Measure; CONUT, Controlling Nutritional Status.

Assigning mean values respectively to age, sex (0: male, 1: female) and PD severity (0: not severe, 1: severe) in the best fitted model the estimated relationship of the CONUT score and FIM gain may be illustrated in Fig 3. The fixed effect was 1.465 for a CONUT score ≤3 and -2.054 for a CONUT score >3, with a random effect of 1. Fig 3 and Table 3 reveal that while FIM gain shows no significant decrease when the CONUT scores are ≤3 (slope coefficient, 1.465; p = 0.183), it decreases significantly when the CONUT scores are >3 (slope coefficient, -2.054; p = 0.026).

## Discussion

Fig 3 shows that the straight line of the FIM gain appears to be increasing if the CONUT score is ≤3, but this is not significant (p = 0.183); the slope of the straight line drastically changes from positive to negative at a CONUT score of 3, and after the score, the FIM gain decreases significantly with an increase in the CONUT scores (p = 0.026). Therefore, a CONUT score of 3 is the change point from which the ADL of patients with PD decreases significantly and monotonically as the score increases. Although these findings appear reasonable, they are only from a retrospective cohort study with a relatively small sample size. Hence, follow-up studies are needed to confirm these findings before using them for developing any nutritional intervention programs to improve the ADL in patients with PD.

The CONUT method, presented at the European Society for Clinical Nutrition and Metabolism in 2002, is a nutritional assessment method that scores the Alb, TLC, and T-cho values. Alb is an indicator of protein metabolism, TLC is an indicator of immunity, and T-cho is an indicator of lipid metabolism, which reflects the systemic condition of the patient. These measures are easily available from the medical records of patients with PD. Tools often used for detecting malnutrition of the elderly are questionnaires or scores based on physical evaluations. However, questionnaires were not considered reliable sources of information from patients with PD. Physical evaluations reflect the consequences of poor nutritional status for a certain period of time, but we wished to apply a tool to detect poor nutritional status as early as possible at the beginning stage, because our ultimate goal was to develop nutritional intervention programs for patients with PD. We found that the CONUT score was an objective and useful measure for this purpose. The association between nutritional status and functional outcomes, obtained using the CONUT method, has been reported in stroke patients [19], gastric cancer [20], hepatocellular carcinoma [21], and heart failure [22].

Four variables, namely, PD severity, dysphagia, cognitive FIM, and grip strength, were identified as confounders. Among them, we selected PD severity as a representative confounder and used it to adjust for confounding. Alternatively, we could have used the propensity score that combines all the confounders into one measure, but the result would not have changed because the four confounders were strongly related to each other, as illustrated in Fig 2.

**Fig 4:**
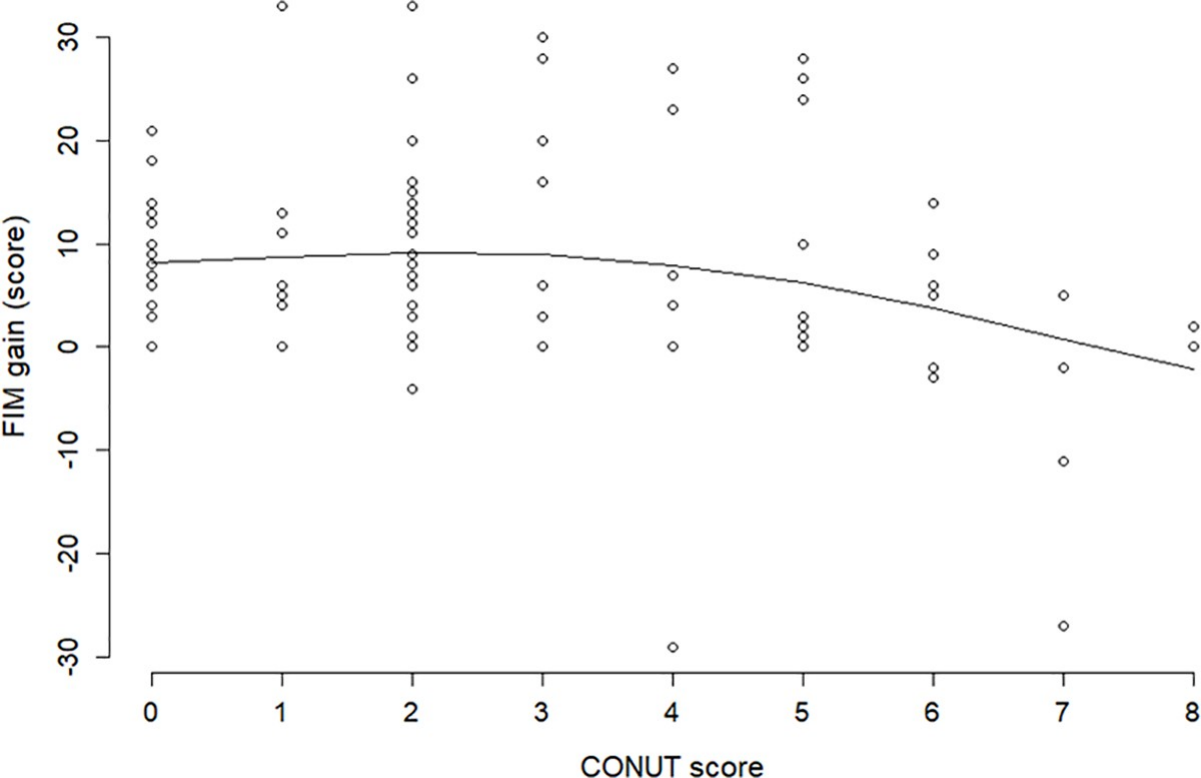
Smoothing spline regression of FIM gain on the CONUT score. Visual inspection of scatter plots between FIM gain and the CONUT score suggested a nonlinear relationship. FIM, Functional Independence Measure; CONUT, Controlling Nutritional Status.

In advanced diseases such as PD, the same patient may be hospitalized multiple times during the progression of symptoms, which may generate highly unbalanced data due to the unequal number of admissions. Therefore, the mixed-effects model was employed to account for within-patient correlation introduced by repeated measurements. This model is often used to correct for individual patient differences in repeated measures [23, 24]. Fig 3 shows that the fitting of the model to data is fairly good. For extra caution, we estimated the relationship between the CONUT score and FIM gain by applying smoothing spline regression that was a data-dependent method, which had much weaker restrictions than the piecewise linear mixed-effects models. The result is illustrated in Fig 4. The comparison of the figure with Fig 3 shows the smoothing spline and piecewise line with knot = 3 grasp with the same tendency of the data; this verifies the validity of the statistical approach of this study.

This study has several limitations. First, the CONUT score was computed from clinical data obtained at an examination point and its variability was not assessed. Second, the sample size of this retrospective cohort study was relatively small. Third, the PD population is highly heterogeneous, and some important confounding factors could have been missed. Further prospective cohort studies with larger sample sizes are needed to confirm the findings of this study.

## Conclusions

The association between nutritional status and ADL is unclear among patients with PD. On employing the CONUT score to measure nutritional status and the motor FIM gain for ADL, and applying sophisticated statistical techniques, we found that a poor nutritional status (i.e., CONUT score >3) was significantly associated with a poor ADL in patients with PD. The finding is based on a retrospective cohort study with a relatively small sample size. Thus, follow-up studies are needed to confirm the finding.

## Supporting information

S1 File Piecewise linear mixed-effects models. (DOCX)

S1 Table Background factors of patients with PD. (PDF)
