# Supplementary material for: Nutritional status and activities of daily living in patients with Parkinson’s disease
Source: PLoS One. 2021 Feb 2;16(2):e0246329. doi: 10.1371/journal.pone.0246329 (PMC7853475; doi:10.1371/journal.pone.0246329)
Supplement: S1 File — (DOCX) [file pone.0246329.s001.docx]

**S1 File: Piecewise linear mixed-effects models.**

Let $y_{ij}$ be FIM gain, $x_{ij}$ be CONUT score, $w_{1ij}$ be age, $w_{2ij}$ be sex, and $w_{3ij}$ be PD severity for episode $j$ of patient $i,$ where $w_{2ij}=\left\{ \begin{matrix} 0 :\mathrm{male} \\ 1 :female \end{matrix} \right.$ and $w_{3ij}=\left\{ \begin{matrix} 0 :not severe \\ 1 :severe \end{matrix} \right.$.

The piecewise linear model with knot=c, which is employed in the study to describe the relationship between FIM gain and the CONUT score, is given as follows:

$y_{ij}=\beta_{0}+\beta_{1}x_{ij}+\beta_{2}{{(x}_{ij}-c)}_{+}+\beta_{3}w_{1ij}{+\beta_{4}w_{2ij}+\beta_{5}w_{3ij}+b}_{i}+\varepsilon_{ij}$,

where ${(x-c)}_{+}\equiv0$, $max(0,x-c)$, $\varepsilon_{ij}\perp b_{i}$, $b_{i}\sim N(0,\sigma_{b}^{2})$, $\varepsilon_{ij}\sim N(0,\sigma_{\varepsilon}^{2})$.

When $x_{ij}\leq c, y_{ij}=\beta_{0}+\beta_{1}x_{ij}+\beta_{3}w_{1ij}{+\beta_{4}w_{2ij}+\beta_{5}w_{3ij}+b}_{i}+\varepsilon_{ij}$,

and when $x_{ij}>c, y_{ij}=\left( \beta_{0}-{c\beta}_{2} \right)+{(\beta}_{1}+\beta_{2})x_{ij}+\beta_{3}w_{1ij}{+\beta_{4}w_{2ij}+\beta_{5}w_{3ij}+b}_{i}+\varepsilon_{ij}$.

Taking each CONUT score as the value of the knot, that is, for each c=1, 2, 3, 4, 5, 6, and 7, we estimate the unknown parameters in the above model, and then compute Akaike’s information criteria (AIC). Moreover, the linear model without knot, that is, the model obtained by putting $\beta_{2}=0$ in the above model, is fitted to the data. The table below gives the values of AIC for each piecewise linear model and the linear model without knot.

**S1 Table. Values of AIC.**

| Model | Piecewise linear  model | | | | | | | Linear model^a^ |
| --- | --- | --- | --- | --- | --- | --- | --- | --- |
| Knot (c) | 1 | 2 | 3 | 4 | 5 | 6 | 7 | ~~-~~ |
| AIC^b^ | 691.0 | 692.0 | 690.7 | 691.7 | 691.5 | 691.6 | 692.1 | 698.0 |

^a^linear model without knot, ^b^Akaike’s information criteria.

The model that achieved the smallest AIC, namely, the piecewise linear model with knot=3, is selected as the best fitted model.
