# Supplementary material for: Nutritional status and activities of daily living in patients with Parkinson’s disease
Source: PLoS One. 2021 Feb 2;16(2):e0246329. doi: 10.1371/journal.pone.0246329 (PMC7853475; doi:10.1371/journal.pone.0246329)
Supplement: S1 Table — (PDF) [file pone.0246329.s002.pdf]

S1 Table: Background factors of patients with PD.

No.	ID	Age	Sex	Number of days hospitalization	Route of hospitalization	Alb	TLC	T-cho	Conut	BMI	Weight loss rate	PD severity	Dysphagia	Grip strength	Adjusting anti-Parkinsonian drugs	Total FIM	Motor FIM	Cognitive FIM	FIM gain
1	1	78	0	48	Home	4	742.5	146	4	27.9	-	0	0	26	0	108	73	35	4
2	1	78	0	31	Acute care hospital	4.1	1344.6	145	2	26.4	1.1	0	0	33.7	0	105	70	35	6
3	1	79	0	32	Home	4.4	1223.6	144	2	25.5	-	1	0	36.7	0	112	77	35	0
4	2	72	0	121	Acute care hospital	2.8	1801.8	151	5	21.7	-5.2	1	1	0	0	22	13	9	0
5	3	79	1	17	Acute care hospital	3.5	2433.9	187	0	20.3	1.2	0	0	15.6	0	117	82	35	6
6	4	83	0	67	Home	3.8	1298	157	2	22.7	1.4	0	0	24.5	0	81	57	24	15
7	5	82	1	57	Acute care hospital	4	1333.3	168	2	19.1	0.6	0	0	14	0	98	63	35	20
8	5	86	1	183	Home	2.8	1025.1	159	7	16.2	-8.4	1	1	9.3	1	85	50	35	-27
9	5	87	1	79	Acute care hospital	2.7	1181.4	126	8	17	-19.5	1	1	0	0	25	13	12	0
10	6	61	0	31	Home	4	2597.4	181	0	23.9	2.7	0	0	46	0	123	88	35	0
11	6	62	0	45	Home	4.3	2860.8	229	0	24.5	3.9	0	0	47.7	0	108	73	35	14
12	6	62	0	33	Home	4.1	1837.5	195	0	23.9	3.4	0	0	46.2	0	122	87	35	0
13	6	63	0	30	Home	4.2	2141.2	197	0	24.2	2.2	0	0	47	0	103	70	33	13
14	6	64	0	49	Home	4	3182	200	0	24.8	1.9	0	0	41.4	0	119	85	34	4
15	6	65	0	29	Home	4.1	1803.2	208	0	25.1	3.4	0	0	45.1	1	111	76	35	6
16	6	66	0	137	Acute care hospital	3.8	1782	194	0	23.6	0.7	1	1	40.6	0	98	68	30	8
17	6	66	0	66	Home	4	2633.1	236	0	25.8	3.3	1	1	35.1	1	102	72	30	7
18	7	87	1	173	Home	2.8	772.2	189	7	14	1.8	1	1	8.9	1	84	49	35	-11
19	8	77	0	163	Acute care hospital	4.1	1468.8	156	2	22.5	14.3	1	1	36.5	1	95	71	24	14
20	8	80	0	150	Acute care hospital	2.7	1484.2	142	6	18	16.9	1	1	12	0	34	15	19	-2
21	9	64	1	68	Acute care hospital	4.3	1781	206	0	25.6	1.2	0	0	23	1	102	67	35	21
22	10	77	0	72	Home	3.1	1455.5	138	5	17	-5	0	1	24.2	0	109	76	33	10
23	11	73	1	62	Acute care hospital	3.4	1012	165	5	23.9	7.3	0	0	18.1	0	89	58	31	24
24	12	85	1	127	Home	3.9	1634	124	2	21.4	0	1	1	15.9	0	95	65	30	4
25	13	76	1	43	Home	3.9	1890	172	1	22.9	0.9	0	0	13.7	0	88	55	33	11
26	14	80	1	113	Acute care hospital	3	1954.8	152	3	16.6	-5.7	1	1	7	0	24	13	11	6
27	14	80	1	23	Acute care hospital	3.3	2678.4	173	3	17.6	0	1	1	7.2	0	24	15	9	0
28	15	80	1	156	Acute care hospital	3.5	4095	169	1	15.7	-5.8	0	1	14.6	0	82	47	35	33
29	16	76	0	106	Acute care hospital	3.8	1229.9	128	3	21.9	1.4	0	1	27	0	77	42	35	20
30	17	70	1	182	Acute care hospital	2.6	1544.4	129	7	16.1	-14.1	1	1	16.3	0	31	20	11	-2
31	17	71	1	146	Acute care hospital	2.6	1060.2	111	8	17.9	4.3	1	1	5	0	21	14	7	2
32	18	77	0	42	Home	3.7	1710	182	0	24.2	4	0	1	35.6	0	101	68	33	0
33	19	77	1	175	Acute care hospital	3	475.2	151	6	16	-18.4	1	1	12	0	34	18	16	-3
34	19	77	1	47	Acute care hospital	3.3	1014.3	181	4	18.6	-4.9	1	1	0	0	21	13	8	0
35	20	61	0	113	Acute care hospital	3.7	1880	128	2	33.7	12	1	0	34	1	91	56	35	11
36	20	61	0	61	Home	4.3	2057.4	139	2	27.7	9.5	1	1	26.9	1	80	51	29	13
37	20	63	0	69	Home	3.7	1662.5	141	1	25.8	7.8	1	1	25.5	0	75	42	33	5
38	21	72	1	194	Other	4.4	1177.4	169	3	22	12.3	1	1	12.4	1	84	64	20	0
39	22	70	0	118	Acute care hospital	4.1	1604.3	177	1	23.4	5	1	0	27.6	1	79	47	32	13
40	23	83	0	69	Other	3.1	2252.8	144	3	20.7	-8	0	0	20.7	0	48	38	10	30
41	24	77	0	76	Acute care hospital	3	1543.3	137	5	22.4	9.4	1	1	6.9	0	30	14	16	0
42	25	79	1	56	Acute care hospital	3.3	2311.5	174	3	22.3	-5.4	1	1	15.9	0	18	13	5	0
43	25	80	1	90	Acute care hospital	3.6	2376.5	161	1	21.7	0	1	1	14.8	0	18	13	5	0
44	26	70	1	46	Home	4.2	1932	298	0	19.1	-1.3	0	0	21.7	0	103	70	33	18
45	27	77	1	121	Acute care hospital	3.9	1426.8	166	2	15.5	2	0	0	13.7	1	101	66	35	8
46	27	78	1	69	Acute care hospital	4.1	1643	184	0	15.1	1.5	1	0	14.9	1	84	49	35	10
47	28	84	1	134	Acute care hospital	3	964	177	5	20.5	6.9	1	1	2.5	0	23	13	10	2

48	28	85	1	15	Acute care hospital	3.5	1566	150	2	18.1	-	1	1	0	0	18	13	5	0
49	29	67	0	53	Home	4	2016	185	0	18.9	3.8	1	1	30	1	79	54	25	0
50	29	69	0	70	Acute care hospital	3.5	1306.4	151	2	15.9	2.8	1	1	21.4	1	38	24	14	-4
51	29	69	0	120	Acute care hospital	3	1663.8	157	3	16.1	-5.5	1	1	12.8	0	25	14	11	3
52	29	70	0	96	Acute care hospital	2.7	1523.2	135	7	15.1	-4.7	1	1	15.2	0	24	13	11	5
53	29	70	0	29	Acute care hospital	2.9	1812.6	134	6	15.5	-3.5	1	1	13.3	0	26	14	12	6
54	30	82	1	197	Acute care hospital	3	1107.6	159	5	14.4	-23.7	0	0	20.7	1	78	51	27	28
55	31	71	0	63	Home	3.8	1989.9	186	0	21.2	-0.3	1	1	0	0	18	13	5	0
56	31	72	0	125	Home	3.1	2026.2	166	3	19.7	-6	1	1	0	0	18	13	5	0
57	31	73	0	37	Home	3.3	2040	161	3	18.9	-4.9	1	1	0	0	18	13	5	0
58	31	73	0	131	Home	3.6	2095.5	170	1	18.9	-11.1	1	1	0	0	18	13	5	0
59	31	74	0	65	Home	3.2	2027.4	158	3	20.1	-5.8	1	1	0	1	18	13	5	0
60	32	70	0	233	Acute care hospital	3.2	1883.6	103	4	22.8	18.3	0	0	27.1	1	87	53	34	23
61	33	79	0	283	Acute care hospital	3.8	2084.4	138	2	21.2	13.7	1	0	16.4	1	45	29	16	9
62	34	72	1	345	Acute care hospital	3.4	1597.2	145	4	19.2	11.9	1	1	15.5	1	71	42	29	-29
63	35	78	0	161	Acute care hospital	3.2	1024.1	159	5	19.3	-7.9	0	1	26.7	0	88	62	26	1
64	35	78	0	195	Acute care hospital	3.7	1421	152	2	19.6	-7	0	1	26.7	1	88	55	33	1
65	35	80	0	48	Home	3.7	1596	144	2	20.4	4.7	1	1	33.1	0	82	52	30	4
66	36	60	1	52	Acute care hospital	4.3	1193.4	168	3	21.4	1.3	0	0	25	0	110	75	35	16
67	37	82	1	128	Acute care hospital	3.4	1353	200	3	15.8	-2.4	1	1	10.3	0	63	35	28	16
68	38	60	1	39	Home	3.9	1507.5	148	2	21.7	1.7	0	0	17.7	1	104	70	34	16
69	38	61	1	44	Home	3.7	1489.2	155	2	20.7	-3.3	0	0	19.9	0	121	86	35	0
70	39	74	1	82	Home	3.8	1501.5	172	2	17.2	-10.3	0	0	23	0	87	57	30	7
71	39	76	1	250	Acute care hospital	3	959.4	168	5	16.2	-6.5	0	1	18	0	47	25	22	26
72	40	70	1	83	Acute care hospital	3.6	1655.4	181	0	29.5	5.8	0	0	21.7	1	102	69	33	12
73	41	86	0	31	Acute care hospital	3.7	507.4	137	5	18.4	1.9	0	0	24	0	76	54	22	0
74	42	73	0	93	Acute care hospital	3.7	2538.9	180	0	21.9	-7.4	0	0	33	0	115	88	27	3
75	43	75	0	20	Acute care hospital	3.8	1368.4	169	2	17.9	-1.7	0	1	18	0	30	18	12	6
76	44	63	0	50	Home	4.2	1696.5	203	0	24.4	3.8	0	0	31	0	100	73	27	9
77	45	79	0	107	Acute care hospital	2.7	1687.8	133	6	15	-15.8	1	1	24	1	51	26	25	5
78	45	79	0	178	Acute care hospital	2.6	1339.5	140	6	15.3	-10.5	1	1	24	1	40	21	19	9
79	46	60	1	175	Home	3.9	1140	214	2	27.3	1	0	0	18.4	1	118	83	35	3
80	47	81	0	99	Acute care hospital	3.6	1305	179	2	18.3	-21.9	1	1	17.9	0	59	29	30	26
81	47	83	0	217	Other	3.1	949	132	6	18.4	-8.7	1	1	21.1	1	29	13	16	14
82	48	80	1	63	Home	3.2	1023	196	4	22.7	1.6	0	1	16.3	0	93	58	35	27
83	49	86	0	61	Acute care hospital	3.2	1962	146	3	18	1.9	1	1	15	1	29	14	15	0
84	50	79	1	211	Acute care hospital	3.9	1725.3	167	1	15.1	-4.2	1	1	13.4	0	39	13	26	5
85	51	72	1	155	Acute care hospital	3.8	1218	244	1	14	2.1	1	1	9.9	0	41	27	14	6
86	52	79	1	109	Acute care hospital	3.6	1303.9	145	2	16.9	-9	1	0	15.8	1	62	40	22	3
87	53	72	1	64	Acute care hospital	3.5	1303.8	149	2	15.2	-4.2	0	1	18.5	0	78	53	25	33
88	54	88	1	71	Acute care hospital	3.2	1950	167	3	19.2	-4.8	0	0	16.3	0	43	30	13	28
89	55	79	1	101	Acute care hospital	3.4	1914	133	4	22.8	-3.5	1	1	10.7	0	37	15	22	7
90	56	75	0	154	Acute care hospital	2.9	4569.5	110	6	17.6	-0.8	1	0	15	1	82	47	35	6
91	56	78	0	82	Home	3.4	1547.7	106	5	18.9	2.9	1	1	13.4	0	66	34	32	3
92	57	47	0	78	Acute care hospital	4.1	1177.6	164	3	18.6	-3.4	0	0	40	1	102	70	32	16
93	58	77	1	157	Acute care hospital	3.6	860.2	161	3	18.8	-5.2	1	0	12.3	0	61	32	29	28
94	59	83	0	31	Acute care hospital	3.9	2917.2	163	1	20.4	1.1	0	0	28	0	117	82	35	4
95	60	69	1	110	Home	3.7	1385.8	169	2	18.9	8.2	0	0	17	1	96	61	35	12
96	61	77	0	81	Acute care hospital	3.6	1365.3	169	2	22.3	-1.1	0	0	33	0	100	65	35	16
